# Mentoring Medical Students Towards Oncology: Results from a Pilot Multi-institutional Mentorship Programme

**DOI:** 10.1007/s13187-020-01919-7

**Published:** 2020-11-26

**Authors:** Kathrine S. Rallis, Anna Wozniak, Sara Hui, Adam Stammer, Cigdem Cinar, Min Sun, Taylor Fulton-Ward, Alison A. Clarke, Savvas Papagrigoriadis, Apostolos Papalois, Michail Ch. Sideris

**Affiliations:** 1 Barts Cancer Institute, Queen Mary University of London, London, UK; 2 Barts and The London School of Medicine and Dentistry, Queen Mary University of London, London, UK; 3 Birmingham Medical School, University of Birmingham, Birmingham, UK; 4 Guy’s, King’s and St Thomas’ School of Medicine, King’s College London, London, UK; 5 International Society for Pelvic Surgery, Athens, Greece; 6 Experimental Educational and Research Centre ELPEN, Athens, Greece; 7 Women’s Health Research Unit, Queen Mary University of London, London, UK

**Keywords:** UK medical students, Mentoring, Undergraduate oncology society, Undergraduate medical education, Undergraduate oncology teaching, Surveys and questionnaires

## Abstract

The mounting global cancer burden has generated an increasing demand for oncologists to join the workforce. Yet, students report limited oncology exposure in undergraduate medical curricula, while undergraduate oncology mentorships remain underutilised. We established an undergraduate oncology society–led mentorship programme aimed at medical students across several UK universities to increase medical student oncology exposure. We electronically recruited and paired oncologist mentors and medical student mentees and distributed a dedicated questionnaire (pre- and post-mentorship) to compare mentees’ self-reported cancer specialty knowledge and oncology career motivation after undertaking a 6-week mentorship. We also determined students’ interest across specialties and subspecialties and measured mentor availability via percentage programme uptake. Statistical analysis included univariate inferential tests on SPSS software. Twentynine (23.4%) of 124 oncology specialists agreed to become mentors. The mentorship was completed by 30 students across three medical schools: 16 (53.3%) Barts, 10 (33.3%) Birmingham, and 4 (13.3%) King’s; 11 (36.7%) mentored by medical oncologists, 10 (33.3%) by clinical/radiation oncologists, and 9 (30%) by surgical oncologists. The mentorship generated a statically significant increase in students’ knowledge of the multidisciplinary team and all oncology-related specialties including academia/research but not interest towards a career in oncology. Undergraduate oncology mentoring is an effective educational, networking and motivational tool for medical students. Student societies are a valuable asset in cultivating medical student oncology interest by connecting students to faculty and increasing mentor accessibility. Further research should focus on developing an optimal mentorship structure and evaluating long-term outcomes of such educational initiatives.

**Supplementary Information:**

The online version contains supplementary material available at 10.1007/s13187-020-01919-7.

## Background

Cancer remains a leading cause of premature death in the UK with one in two born after 1960 expected to be diagnosed with cancer during their lifetime [1]. The emerging cancer burden has inevitably exerted substantial strain on the United Kingdom (UK) National Health Service (NHS) generating an increasing demand for oncologist to join the workforce. This growing demand for oncologists has largely exceeded supply of trainees, evident by the threefold increase in vacant clinical oncologist consultant posts in 2017 [2]. Indeed, the total number of trainees predicted to enter the workforce in the next 5 years will not fill these vacant posts, a concerning figure attesting to the necessity for further action [2, 3].

Meanwhile, studies globally report limited exposure to oncology specialties in undergraduate medical education curricula [4–7], student teaching dissatisfaction with their oncology education [8] and lack of confidence with oncology care [4, 9, 10]. Furthermore, although medical mentorships confer notable benefits for both mentors and mentees, and are an established medical educational tool at trainee level [11–15], mentorships for undergraduate medical students are limited and predominantly restricted to surgery, general medicine and emergency medicine [16–18].

Notably, student interest groups, also known as undergraduate student societies, have been shown to offer valuable benefits in fostering early career interest by building student-faculty mentorship relations and encouraging field-specific research, as demonstrated by their effectiveness across several medical specialties including oncology [16, 18–24]. Nevertheless, further work is necessary to connect medical students to oncology faculty mentors [24].

Therefore, we aimed to increase medical students’ exposure to oncology specialties, including medical, clinical/radiation and surgical oncology, through a 6-week undergraduate oncology society–led mentorship programme aimed at both pre-clinical and clinical year medical students across several UK universities.

## Methods

### Participants

#### Undergraduate Oncology Societies

Following an initial pilot mentorship programme set-up by Barts and The London (BL) Oncology Society in January 2019, all known undergraduate oncology societies in UK medical schools, equivalent to student oncology interest groups reported in Canadian and USA medical schools, were contacted via social media and email, and invited to set-up a mentorship programme at their university (September 2019). Where a medical school did not have an established undergraduate oncology society, an undergraduate medical society or medical student representative was contacted instead.

#### Mentors

Medical, clinical/radiation and surgical oncologists who had completed their specialty training and were senior registrars, consultants or academics based at university teaching hospitals or research institutes were identified via online search of NHS Trust and UK Cancer Institute staff directories, few recommended by personal affiliation, and contacted by email (Appendix 1) requesting their participation as mentors and outlining the potential environments (clinic, hospital and research setting) and domains (medical oncology, clinical oncology, surgical oncology and academic/clinical research oncology) in which mentees and mentors could engage in. Of those agreeing to participate, we requested their weekly timetable availability for distribution to their allocated mentee.

#### Mentees

Oncology societies advertised the mentorship programme to medical students at their university via mailing list and social media as well as formal university channels including year groups and newsletter. Electronic applications were received over a 10-day period. Successful applicants were determined by ranking according to objective scoring of de-identified personal statements by two independent student coordinators. A maximum of five points was allocated in each of the four domains including (a) insight into oncology career, (b) motivation, (c) previous experience and (d) signs of interest. Applicants with the highest sum of scores across both assessors (maximum 40 points) secured a mentor until no more places were left. The applicants who ranked lower and did not secure a mentor were not accepted onto the programme.

### Mentorship (Intervention)

**Fig. 1:**
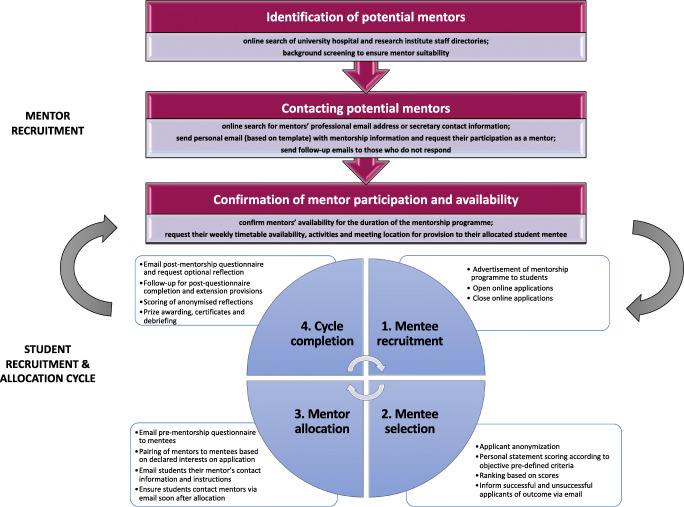
Structured model for setting up a student mentorship programme through a student interest group outlining the process of mentor recruitment and the student allocation cycle

Each mentorship cycle ran over approximately 6 weeks during which period mentees were instructed to meet their mentor on at least three occasions to attain a certificate of completion. Mentees were required to submit an electronic pre-mentorship questionnaire prior to receiving their mentors’ contact details. Mentor-mentee pairing was determined by mentor availability and students’ declared interest on their application. Mentees were emailed their mentors’ contact information, career description, timetable availability and hospital location with further instructions. Students were encouraged to reflect on their experience and submit an optional reflective piece for a chance at winning a book-prize award. The instructions provided to mentors and mentees (Appendix 1 and 2, respectively) were the same across all sites and specialties and ensured a relative uniformity of placements. These explained the aim of the programme, expectations, timeline and offered examples of settings in which students could shadow mentors. There was no strict curriculum as we endeavoured to allow flexibility for students to direct their learning according to their interests and aimed to tailor opportunities across the different sites. This primarily depended on mentor allocation and local facilities. The post-mentorship questionnaire was disseminated to student mentees at the end of the 6-week period. Students who did not complete the post-mentorship questionnaire were personally contacted up to three times via email to enquire why they had not done so. An extension was given to any student requiring additional time to meet their mentor. A new allocation cycle was to commence when students had finished their 6-week mentorship and mentors had become once again available for allocation to a subsequent pool of student mentee applicants (Fig. 1).

### Questionnaires (Intervention)

Questionnaires were designed and compared with available ones in the literature. Discussions between research team members contributed to the final questionnaire components and design. The pre- and post-mentorship questionnaires (Appendix 3 and 4, respectively) assessed students’ responses in two domains including their perceived oncology-related interest and knowledge, while a third and fourth section focused on reflection and feedback. The pre-mentorship questionnaire compromised of nine questions: six on a five-point Likert scale, two multiple choice and one free text response; whereas the post-mentorship questionnaire compromised of 17 questions: seven on a five-point Likert scale: six identical to pre-mentorship questionnaires, four multiple choice and six free text response. The sign-up application (Appendix 2) gathered information pertaining to students’ demographics, including their year of study, as well as their oncological specialty and subject of interest using multiple choice and free response questions.

### Comparisons

We compared responses across student demographics including gender, medical school and year of study. We also compared responses across different specialty of interest (i.e. medical, clinical/radiation or surgical oncology) and subject (e.g. gastrointestinal, gynaecological, haematological cancers etc.) of mentoring placement. Finally, we compared students’ perceived oncology-related interest and knowledge pre- and post- mentorship.

### Outcomes

The effectiveness of the mentorship programme as an educational, networking and motivational tool was measured by assessing for a significant change (*p* < 0.05) in students’ pre-/post-mentorship responses to Likert scale questions in addition to evaluating free text responses.

### Procedures to Minimize Bias

Questionnaires were piloted in a small group of medical students before being reviewed and launched to ensure questions were unambiguous and phrasing did not generate responder bias.

### Data Collection

All surveys were conducted via a standardised, web-based, data collection form (Google Form, Google, Alphabet Inc.). Responses were saved to a password-protected Excel file. Regarding free text responses (qualitative data), K.R. proceeded to a pilot thematic analysis; this resulted in discrete thematic axes. These thematic axes were revised by the senior author of the study to ensure data accuracy and any discrepancy was resolved through discussion.

### Statistical Analysis

Statistical analysis was completed on IBM SPSS Statistics software for Mac, Version 26. Descriptive statistics were used to analyse demographics, student interest and feedback. Following assessment of data distributions, we used Wilcoxon signed-rank (WSR) test for paired associations supplemented by Mann-Whitney *U* (MWU) test for ineffectively paired groups as determined by Spearman correlation coefficient (one-tailed). *p* value less than 0.05 was considered as statistically significant.

## Results

### Demographics

#### Undergraduate Oncology Societies

Forty-two medical schools, including 20 undergraduate oncology societies, were contacted for joining the study (Supplementary Table 1). In addition to BL, five undergraduate oncology societies agreed to participate, though only two successfully established a mentorship programme including Kings College London (KCL) and University of Birmingham Medical School (UBMS) Oncology Societies. The three societies that did not succeed in establishing a mentor programme either withdrew interest due to time restraints of committee members, postponed the programme for the next academic year or cancelled the programme before it began due to coronavirus disease 2019 (COVID-19) lockdown measures and concerns. Three mentorship allocation cycles were carried out successfully at BL, one at KCL and one at UBMS.

#### Mentors

Out of a total of 124 potential mentors contacted and asked to participate in the mentorship programme across all three NHS Foundation Trusts, only 29 (23.4%) agreed to participate. Table 1 shows a breakdown of mentors’ specialties, subspecialties and NHS Trust affiliation.

**Table 1:** NHS Trust affiliation, specialty and subspecialty of all contacted (*n* = 124) and confirmed (*n* = 29) mentors

	Contacted Mentors^a^	Confirmed Mentors^a^
NHS Foundation Trust		
Barts Health	36 (29.0)	12 (41.4)
University Hospitals Birmingham	49 (39.5)	13 (44.8)
King’s College Hospital	39 (31.5)	4 (13.8)
Specialty		
Medical Oncology	56 (45.5)	11 (37.9)
Clinical/Radiation Oncology	29 (23.6)	8 (27.6)
Surgical Oncology	31 (24.4)	10 (34.5)
Other*	7 (5.7)	–
Subspecialty^b^		
Bone cancer	1 (0.8)	–
Brain cancer	5 (4.0)	2 (6.9)
Breast cancer	40 (32.3)	7 (24.1)
Colorectal cancer	13 (10.5)	6 (20.7)
Gastrointestinal cancer	21 (16.9)	4 (13.8)
Germ cell tumours	3 (2.4)	–
Gynaecological cancer	15 (12.1)	3 (10.4)
Haematological cancer	7 (5.7)	1 (3.5)
Head and neck cancer	8 (6.5)	–
Hepatobiliary cancer	8 (6.5)	1 (3.5)
Lung cancer	26 (21.0)	6 (20.7)
Lymphoma	11 (8.9)	3 (10.4)
Melanoma	4 (3.2)	2 (6.9)
Neuroendrocine cancer	5 (4.0)	2 (6.9)
Neurological cancer	4 (3.2)	–
Paediatric cancer	2 (1.6)	1 (3.5)
Sarcoma	9 (7.3)	5 (17.2)
Skin cancer	9 (7.3)	3 (10.4)
Teenager and young adult cancer	2 (1.6)	–
Urological cancer	31 (25.0)	8 (27.6)

^a^Data given as number of mentors (%)

^b^One or more subspecialties per mentor

^*^Other specialties included cardiology (*n* = 1), haematology (*n* = 3), palliative medicine (*n* = 2) and pneumonology (*n* = 1) cancer specialists

#### Mentees

We received 79 applications out of which 43 (54%) students were accepted onto the programme by ranking high enough to secure a mentor, completing the pre-mentorship questionnaire, and 30 (70%) successfully completed the mentorship, responding to the post-mentorship questionnaire. Table 2 summarises students’ demographics. Eleven (36.7%) students were mentored by medical oncologists, 10 (33.3%) were mentored by clinical/radiation oncologists and 9 (30%) by surgical oncologists. Most students met with their mentor on three occasions which was also the median number of mentor-mentee meetings (IQR = 2).

**Table 2:** University affiliation, year of study and gender of all applicants (*n* = 79) and mentees who completed the mentorship (*n* = 30)

	Total applicants^a^	Mentees^a^
University		
Barts and the London	50 (63.3)	16 (53.3)
Birmingham University	14 (17.7)	10 (33.3)
King’s College London	15 (19.0)	4 (13.3)
Year of study		
Year 1	28 (35.4)	7 (23.3)
Year 2	21 (26.6)	8 (26.7)
Year 3	19 (24.1)	10 (33.3)
Year 4	7 (8.9)	4 (13.3)
Intercalating	4 (5.1)	1 (3.3)
Gender		
Male	24 (30.4)	10 (33.3)
Female	55 (69.6)	20 (66.7)

^a^Data given as number of students (%)

### Student Interest

Table 3 captures students’ self-reported interest in different oncology sub-specialties and fields. Most students were interested in medical oncology (78.5%) and academia/research (68.4%) followed by surgical (64.6%) and clinical/radiation (57%) oncology. The most popular fields of interest were haematological (81%), lung (72.2%) and gastrointestinal cancers (70.9%), while urological cancers (43.0%) gathered the least interest.

**Table 3:** Declared specialty and field of interest of all applicants (*n* = 79) and mentees who completed the mentorship (*n* = 30)

	Total applicants^a^	Mentees^a^
Specialty of interest^b^		
Medical oncology	62 (78.5)	25 (83.3)
Clinical/radiation oncology	45 (57.0)	18 (60.0)
Surgical oncology	51 (64.6)	16 (53.3)
Academia/research in oncology	54 (68.4)	24 (80.0)
Field of interest^b^		
Breast cancers	52 (65.8)	17 (56.7)
Gastrointestinal cancers	56 (70.9)	22 (73.3)
Gynaecological cancers	43 (54.4)	14 (46.7)
Haematological cancers	64 (81.0)	23 (76.7)
Head and neck cancers	44 (55.7)	14 (46.7)
Lung cancers	57 (72.2)	22 (73.3)
Urological cancers	34 (43.0)	15 (50.0)

^a^Data given as number of students (%)

^b^One or more selected by students

### Impact of Mentorship Programme

The mentorship programme generated a statistically significant improvement in students’ knowledge of the multidisciplinary team (3.2 vs. 4.0/5, *p* < 0.001) as well as the role of medical (3.1 vs. 4.0/5, *p* < 0.001), surgical (2.8 vs. 3.4/5, *p* = 0.006) and clinical oncologists (2.9 vs. 3.8/5, p < 0.001) and their involvement in academia/research (3.2 vs. 4.0/5, *p* = 0.001) (Table 4). Mentees’ interest in oncology remained unchanged.

**Table 4:** Comparison of mentees’ responses from pre- and post-mentorship questionnaires (*n* = 30)

Question	Pre-mentorship^a^	Post-mentorship^a^	*p* value^b^
Rate your interest in oncology	4.7 ± 0.5	4.7 ± 0.5	0.738
Rate your knowledge of the following:			
Members of the multidisciplinary team in oncology services	3.2 ± 0.6	4.0 ± 0.8	< 0.001*
The role of medical oncologists	3.1 ± 0.8	4.0 ± 0.8	< 0.001*
The role of surgical oncologists	2.8 ± 0.9	3.4 ± 1.1	0.006
The role of clinical oncologists	2.9 ± 0.7	3.8 ± 1.1	< 0.001
The involvement of oncologists in academia/research	3.2 ± 0.8	4.0 ± 0.8	0.001*

^a^Data is reported as the mean value of the Likert score ± standard deviation

^b^*p* value obtained from WSR test analysis between pre- and post-mentorship questionnaires

*Inneffective pairing as determined by Spearman correlation coefficient (one-tailed); *p* value confirmed on MWU test analysis between pre- and post-mentorship questionnaires

### Other Student-Reported Benefits (Qualitative Feedback)

Mentees reported several additional benefits from the mentorship programme in free text responses. We categorised those into certain thematic axes stated on Table 5. Moreover, 28 (93.3%) students believed that this programme has made them a better medical student or future doctor and 29 (96.7%) reported that they would have chosen to do it again.

**Table 5:** Mentees’ free text responses to qualitative feedback in post-mentorship questionnaire (*n* = 30)

Question	Mentees^a^
What is the most important thing you gained from this programme?^b^	
Clinical experience	10 (33.3)
Communication skills	9 (30.0)
Learn about cancer patient management	5 (16.7)
Connect with mentors	4 (13.3)
Familiarise with breaking bad news	4 (13.3)
Insight into multidisciplinary team	4 (13.3)
Insight into research (including clinical trials)	4 (13.3)
Inspired and motivated	3 (10.0)
Observe doctor-patient relationship	3 (10.0)
Research opportunity	3 (10.0)
Academic, research or career advice	3 (10.0)
Insight into oncologists’ work	2 (6.7)
Confirm career aspiration	1 (3.3)
Consolidate textbook learning	1 (3.3)

^a^Data given as number of students (%)

^b^One or more responses per student

## Discussion

### Findings

The mentorship aimed to increase medical students’ exposure of oncology specialties. Results demonstrate a statistically significant increase in students’ self-reported knowledge surrounding all oncology specialties, the multidisciplinary team and cancer research regardless of their allocated mentor’s specialization. Mentees report gaining valuable clinical experience and communication skills by observing the doctor-patient relationship and the breaking of bad news. They also learned more about the management of cancer patients and became inspired and motivated to pursue a career in oncology. Clearly, there is no lack of interest amongst students who are evidently drawn to all oncology specialties including academia and research, and are willing to engage in extracurricular teaching amongst their busy schedules. No increase in student interest in oncology was observed post-mentorship as students who applied for the programme already had a strong interest in oncology from the start. Poor mentor, and placement, availability are significant factors that limit undergraduate medical students’ exposure to oncology as demonstrated by low mentor uptake (23.4%) to participate in this programme which was most pronounced at KCL where only four out of 39 contacted professionals agreed to become mentors. Differences in mentor availability across insitutions could potentially be explained by the variation in service demands in geographical regions, with physicians based at high-demand oncology centres less likely to be able to dedicate time to this type of extracurricular teaching.

### Significance of Findings

The growing global demand for oncologists to join the workforce underscores the necessity of early undergraduate oncology teaching. Nationally, students advocate for more clinical exposure to oncology, increased teaching hours, more diverse coverage of cancer topics and more clinical skills teaching focusing on breaking bad news and communicating with terminally ill patients. Our findings address these issues and are consistent with previous research showing that early mentorships significantly impact career selection, career success, research productivity and student wellbeing whilst improving academic inclusivity of students from traditionally underrepresented backgrounds and narrowing the sex gap [16, 17, 25–32]. We also demonstrate the effectiveness of student-led societies in increasing student accessibility to mentors, ultimately serving as an important motivational, networking and educational resource.

### Recommendations

Our findings testify to the value of undergraduate societies in creating student to faculty connections which in turn improve students’ career prospectus, research productivity, but also their wellbeing, since effective communication skills have been shown to be a cost effective way of preventing physician burnout [33–35]. Increasing students’ oncology exposure is a compelling strategy to prevent specialty attrition by inspiring a future generation of holistically qualified oncologists who are interested in spearheading laboratory and clinical research innovations [36–38]. Hence, we advocate for more widespread adoption and proactive use of student-led oncology societies in UK medical schools. Agarwal et al. at Boston University School of Medicine, provides a detailed model for other medical schools to initiate their own student oncology societies [24]. Further to this, our report provides a structured model for setting up mentorship programmes through student interest groups (Fig. 1).

### Strengths

To our knowledge, this is the first UK-reported data on the value of oncology mentoring for undergraduate medical students in addition to being the first UK report of an undergraduate oncology society–led mentorship initiative. The strengths of this study include the multi-institutional representability of findings across several medical schools, and the variety of oncological specialties and subspecialties examined, providing valuable insight into students’ interests and the benefits of oncology mentoring in different settings.

### Limitations

Limitations to this study include limited sample size, largely due to restricted mentor availability. Also, those are results from a pilot study where questionnaire validation was not possible. Increases in students’ self-reported knowledge of oncology post mentorship, albeit corroborated by qualitative feedback to open response questions, was not validated by an objective method of assessment and therefore positive changes could have been influenced by response acquiescence or acceptance bias which lead students to provide a higher estimate of their knowledge in the post-mentorship questionnaire.

### Future Endeaveours

We plan to introduce this mentorship programme nationally across all UK universities. This would allow a larger sample size and increase the amplitude of our results, allowing sub-group analysis by university, allocated mentorship specialty and subspecialty. Organising 3 mentorship cycles per academic year at each university would also aid in increasing mentee capacity. Enabling students to rotate on several specialties would provide a more holistic experience, while providing students with an outcome-based logbook would benefit them in structuring their learning. Extending students’ placement is also desirable according to mentees’ feedback in free text responses. Measuring students’ knowledge by an objective assessment, such as a multiple-choice question test, before and after the programme would have been a better means to assess improvements in oncology knowledge. Long-term effects of such mentorship programmes on influencing students’ specialty selection can be investigated by following up mentees’ specialty training pathway in the future. Further research into understanding the motivations of mentors, their reasons for participating in the programme as well as any issues that may discourage their participation may help address the obstacles faced with mentor recruitment and allow for broadening of the programme if more mentors can be recruited.

## Conclusion

In summary, findings herein demonstrate the effectiveness of undergraduate oncology mentoring as an educational, networking and motivational tool with medical student mentees reporting a statistically significant increase in self-reported knowledge in all areas of oncology examined. Students’ interest and desire to engage in oncology research and clinical exposure is largely unmet by restricted mentor, and placement, availability. Further efforts should be made to increase oncology-related placement availability, teaching exposure and research opportunities for medical students within undergraduate medical curricula and extracurricular settings. Student societies are a valuable asset in cultivating student interest in oncology due to their ability to connect students to faculty members thus increasing students’ accessibility to mentors. Further research should focus on developing an optimal structure for mentorships and evaluating the long-term outcomes of such educational initiatives.

### Supplementary Information

Table S1 (DOCX 16 kb)

## Appendix 1 Template email for mentor recruitment

Dear Dr. [insert potential mentor’s name],

I am contacting you on behalf of [insert oncology society name], a student-led society for medical students at [insert medical school name]. Our aim is to help medical students engage with oncology and increase awareness of the opportunities for research and clinical practice in this field.

We are organising a mentorship scheme to allow medical students to gain more exposure and insight into oncology from an early stage in their training. Students will be able to shadow oncologists in clinic, hospital and research settings with aim to cover the following four domains:

- Medical oncology
- Clinical oncology
- Surgical oncology
- Academic/clinical research oncology

Students will apply formally for this scheme, applications will be screened, and students will be assigned to mentors based on compatibility of interests.

We would like to ask if you would be willing to take on 1–2 students as your mentees for a period of 6 weeks sometime between [insert months and year over which mentorship cycle with take place]. Mentees and mentors may choose to maintain their working relationship after this 6-week period. We hope interested students may even get the opportunity to participate in research.

If you are interested in being a mentor, please reply to this email and I can forward you further details. We will need your timetable availability for a 6-week period and a contact number for students to get in touch with you.

We would be very grateful for your help in this project.

Please do not hesitate to contact me if you have any questions.

Kind regards,

[insert full name of committee member mentorship coordinator].

[insert role of committee member in named oncology society]
