# Supplementary material for: Mentoring Medical Students Towards Oncology: Results from a Pilot Multi-institutional Mentorship Programme
Source: J Cancer Educ. 2020 Nov 26;37(4):1053–65. doi: 10.1007/s13187-020-01919-7 (PMC7688447; doi:10.1007/s13187-020-01919-7)
Supplement: Supplementary file 1 — (DOCX 16 kb) [file 13187_2020_1919_MOESM1_ESM.docx]

# Supplementary materials

**Supplementary Table 1. UK medical schools invited to participate in the study by setting up a mentorship programme at their institution.**

| Contacted UK Medical Schools |
| --- |
| Aberdeen*^†^ |
| Anglia Ruskin |
| Aston |
| Barts and The London*^†^ |
| Birmingham*^†^ |
| Brighton & Sussex |
| Bristol* |
| Buckingham |
| Cambridge* |
| Cardiff* |
| Dundee |
| Edge Hill |
| Edinburgh* |
| Exeter |
| Glasgow* |
| Hull York |
| Imperial College London* |
| Keele |
| Kent and Medway |
| King's College London*^†^ |
| Lancaster |
| Leeds* |
| Leicester |
| Liverpool* |
| London School of Hygiene & Tropical Medicine |
| Manchester* |
| Newcastle*^†^ |
| Norwich (University Eeast Anglia)* |
| Nottingham |
| Nottingham-Lincoln |
| Oxford* |
| Plymouth University-Peninsula School of Medicine |
| Queen's University Belfast |
| Sheffield* |
| Southampton* |
| St Andrews* |
| St George's University London* |
| Sunderland |
| Swansea |
| University Central Lancashire |
| University College London (UCL)*^†^ |
| Warwick |

* UK medical schools with established undergraduate oncology societies as of September 2019.

^†^ Undergraduate oncology societies agreeing to participate in the study.
